# A novel function for cyclin D1 as a transcriptional role in oncogenesis and tumor development by ChIP-Seq and RNA-Seq

**DOI:** 10.7150/jca.52909

**Published:** 2021-06-26

**Authors:** Yudi Xiong, Yuan Wang, Tianqi Li, Xiaoyan Yu, Yangyang Zeng, Guohui Xiao, Fuxiang Zhou, Yunfeng Zhou

**Affiliations:** 1 Hubei Cancer Clinical Study Center, Hubei Key Laboratory of Tumor Biological Behaviors, Zhongnan Hospital, Wuhan University, Wuhan, China.; 2 Department of Radiation Oncology and Medical Oncology, Zhongnan Hospital, Wuhan University, Wuhan, China.

**Keywords:** Cyclin D1, Prognosis, Transcriptional role, ChIP-seq, RNA-seq

## Abstract

**Introduction:** Aberrations in cell cycle control is defined as one of the hallmarks of cancer, while cyclin D1 is an essential protein to cell cycle which promote G1 phase into S phase, and frequently overexpressed in many human cancers. However, new functions have been identified in transgenic mice models, including the transcription of genome, the development of chromosome instability and DNA repair. In this research, our aim is to find the function of cyclin D1 in transcription in human cancers.

**Methods:** The correlation of the cyclin D1 expression levels and prognosis of cervical cancer patients were analyzed in tissue microarray (TMA) cohort. We chose C33A as our main research object. Using chromatin immunoprecipitation sequencing (ChIP-seq) coupled with RNA sequencing (RNA-seq), to find out the genes differentially expressed in C33A, cyclin D1 knock-in C33A and cyclin D1 knock-down C33A.

**Results:** We found that upregulation of cyclin D1 was associated with shorter overall survival (OS) and disease-free survival (DFS). Functionally, we identified 422 genes differentially expressed through analysis of the results of ChIP-seq and RNA-seq. These genes are highly enriched in Gene Ontology categories and involve in diverse cellular functions via KEGG classification, including replication and repair, signal transduction, cell growth and death.

**Conclusion:** These findings suggested that the expression of cyclin D1 was associated with the prognosis of patients with cervical cancer. Cyclin D1 can serve both to activate and downregulate gene expression as a transcriptional role directly binding with genome DNA, which means that cyclin D1 may be a key protein during oncogenesis and tumor development.

## Introduction

Cancer has caused more deaths than all coronary heart disease or stroke since from 2011, which is still a major public health problem 1. The cancer burden is increasing these years particularly in low and middle income countries, although many new cancer treatments or strategies are developed and used nowadays. The results show that in 2017 there were 24.5 million incident cases and 9.6 million deaths due to cancer; the incident cases increased by 33% between 2007 and 2017 2. Based on these data, it is necessary to find the factors of leading the tumorigenesis and cancer development. It has been found that aberrations in cell cycle control is defined as one of the hallmarks of cancer 3. Cyclin D1 is a key protein to regulate the cell cycle which promote G1 phase into S phase by binding with CDK4 or CDK6, then phosphorylates and inactivates pRb proteins to regulate nuclear DNA synthesis and mitochondrial biogenesis 4, 5, and frequently overexpressed in many human cancer, such as breast cancer 6, pancreatic cancer 7, colorectal carcinoma 8, lung cancer 9, 10. Scientists designed the CDK4/6 inhibitors to treat cancer based on this target 11-14, but it doesn't work in some tumor patients or cancer cells unexpectedly 15-18. What's more, it was reported that the new functions of cyclin D1 have been identified in transgenic mice models, including the transcription of genome, the development of chromosome instability and DNA repair 19-21. We think cyclin D1 may regulate downstream genes independent of cell cycle, which could be a cause of drug resistance as a transcriptional role.

In order to find the clinic correlation of cyclin D1 and reveal the mechanism of the genome-wide cyclin D1 binding sites and the changes of downstream genes in cancer, we firstly analyzed the correlation between cyclin D1 expression and prognosis in the tissue microarray (TMA) cohort. And then performed the ChIP-seq (Chromatin immunoprecipitation followed by high-throughput sequencing), which is a method to analysis the comprehensive identification of the binding sites of DNA-associating proteins across the genome at high resolution^22^, then established the overexpression and down-regulation cell models, obtained the transcriptomics by RNA-seq. Through analysis of the results of ChIP-seq and RNA-seq, we can study the new function of cyclin D1 protein.

## Materials and Methods

### Tissue microarray

The tissue microarray, purchased from Shanghai Outdo Biotech, contained 126 cervical cancer specimens and 42 paired non-tumor specimens (Outdo cervical cancer cohort, HUteS168Su01). The study protocols were approved by the hospital's research ethics committee and were conducted in accordance with the principles expressed in the Declaration of Helsinki.

### Cell line and cell culture

The human cervical cancer cell line C33A was maintained in our laboratory. The cell line was cultured in DMEM containing 10% fetal bovine serum and incubated in 5% CO_2_ at 37 °C (for more details, please refer to the materials and methods in our other article 17).

### Cell transfection

The plasmids contained the ShRNA duplexes were designed against CCND1 with the following sequences: 5′- ATTGGAATAGCTTCTGGAAT-3′, and the negative control sequences: 5′-AATTCTCCGAACGTGTCACGT-3′. The plasmids containing these sequences were named pLKO.1-Puro-shRNA-CCND1 and pLKO.1-Puro-shRNA.The stable transfection cell lines were named C33A-shNC and C33A-sh, respectively. The plasmids contained the whole CCND1 coding region were named pHAGE-Puro-HA-Flag-CCND1, the negative control plasmids were named pHAGE-Puro-HA-Flag. Therefore, the stable transfection cell lines were named C33A-OENC and C33A-OE. All these plasmids were bought from Bioeagle (Wuhan, China).

### Immunofluorescence assay

The immunofluorescence of cyclin D1 and Flag were used to determine the expression of C33A-shNC and C33A-sh, C33A-OENC and C33A-OE. Cells were grown in 12-well plates and fixed with 4% paraformaldehyde. The cells were incubated with the cyclin D1 primary antibody (1:100 dilution,#A10757, ABclonal, USA) and HA-Tag antibody (1:100 dilution,#AE008, ABclonal, USA) and then incubated with FITC-conjugated donkey anti-rabbit antibody (1:100 dilution, #SA00003-8, Proteintech, China) or CoraLite594-conjugated donkey anti-mouse antibody (1:200 dilution, #SA00013-7, Proteintech, China) for 2h. The cells were imaged on a fluorescence microscope. (For more details, please refer to the materials and methods in our other article 17).

### RNA Extraction and Quantitative Real-Time PCR

Total RNA was isolated from cells using Tirol reagent (Vazyme, China) according to the manufacturer's protocol. Then the cDNA was synthesized from about 1 μg of total RNA by the HiScript^®^ Q RT SuperMix for qPCR (+gDNA wiper) (#R123, Vazyme, China) at 25 °C for 10 min, followed by 50 °C for 30 min, and finally 85 °C for 5 min. Real-time PCR was performed with ChamQ^TM^SYBR^®^qPCR Master Mix(#Q321-02, Vazyme, China) in a 20 μL reaction volume (10 μL SYBR Master Mix, 10 μM forward and reverse primers, 7.2 μL H2O and 1 μL cDNA template) on a CFX Connect™ instrument (Bio-Rad, CA). The human GAPDH gene was performed as an internal control. The following protocol was used for GAPDH and CCND1: preincubated at 95 °C for 30 sec followed by 40 cycles of 95 °C for 5 sec, 58 °C for 15 sec and 72 °C for 30 sec. (For more details, please refer to the materials and methods in our other article 17).

### Western blotting

Protein lysates were extracted from cells treated with PMSF and phosphatase inhibitor tablets. The concentrations of the protein lysates were quantified by BCA assay. These samples were separated by 10% SDS-PAGE and transferred to PVDF membranes which followed with incubated with primary antibodies Cyclin D1 and GAPDH at 4 °C overnight. The membranes were visualized by ECL after the incubation with the secondary antibody. The bands were detected using the ChemiDoc XRS+ system.

### ChIP-seq

ChIP material was performed in accordance with the Magna ChIP (Millipore) manufacturer's guidelines. Briefly, the C33A cells were harvested in 1.5 mL ice-cold PBS and fixed for 15 minutes with 37% paraformaldehyde (final concentration, 1%). 2 mol/L glycine (final concentration, 0.125 mol/L) was added into the unreacted formaldehyde for 10 min. Then washing the cells with ice-cold PBS twice, and the pellets were harvested into PBS (1 ml) with protease inhibitor cocktail and pooled together in a 1.5 mL tube for obtaining the cells. The DNA fragmentation of the pellets was achieved by sonication. IP was performed with 5 uL cyclin D1 antibody (1:5000 dilution, #60186-1-lg, Proteintech, China) and the Input as negative control. Washes and elution of the IP DNA were also prepared according to the Magna ChIP protocol (Millipore). Sample libraries were made by using Rubicon ThruPLEX® DNA-seq Kit and sequenced by using the HiSeq platform (Illumina).

### RNA-seq

RNA extraction, rRNA depletion, library construction, and sequencing were done by mega genomics company. The sequenced Reads were from the HiSeq platform (Illumina) which were saved as FASTQ reads.

### Statistical analyses of ChIP-seq

The raw data was filtered to give high-quality data (clean data) by Trimmomatic (version 0.30). Then, the clean data was mapped with human reference genome-hg 19 by BWA software package. The enrichment interval was obtained by using MACS (version 1.4.2), and the Motif of the enrichment interval was predicted by MEME 23.

### Statistical analyses of RNA-seq

The data was mapped by using HISAT2 24. The raw data was analyzed in R (version 3.5.1). Differential analysis was performed with DESeq 2 25.

### Pathway enrichment analysis

The pathway enrichment analysis for GO and KEGG was performed using the safe package 26.

### Statistical analysis

Differences in clinicopathologic factors between the cyclin D1 high- or low-expression groups were analyzed via the Chi-square test. The Kaplan-Meier and log-rank test methods were used to determine the survival rate. Student's t- test was applied into the analysis of data statistically. It was considered statistically significant that the P value of <0.05. Statistical analyses were performed with IBM SPSS Statistics v20.0 software.

## Results

### Upregulated cyclin D1 expression is associated with poor prognosis in cervical cancer TMA cohort

**Figure 1:**
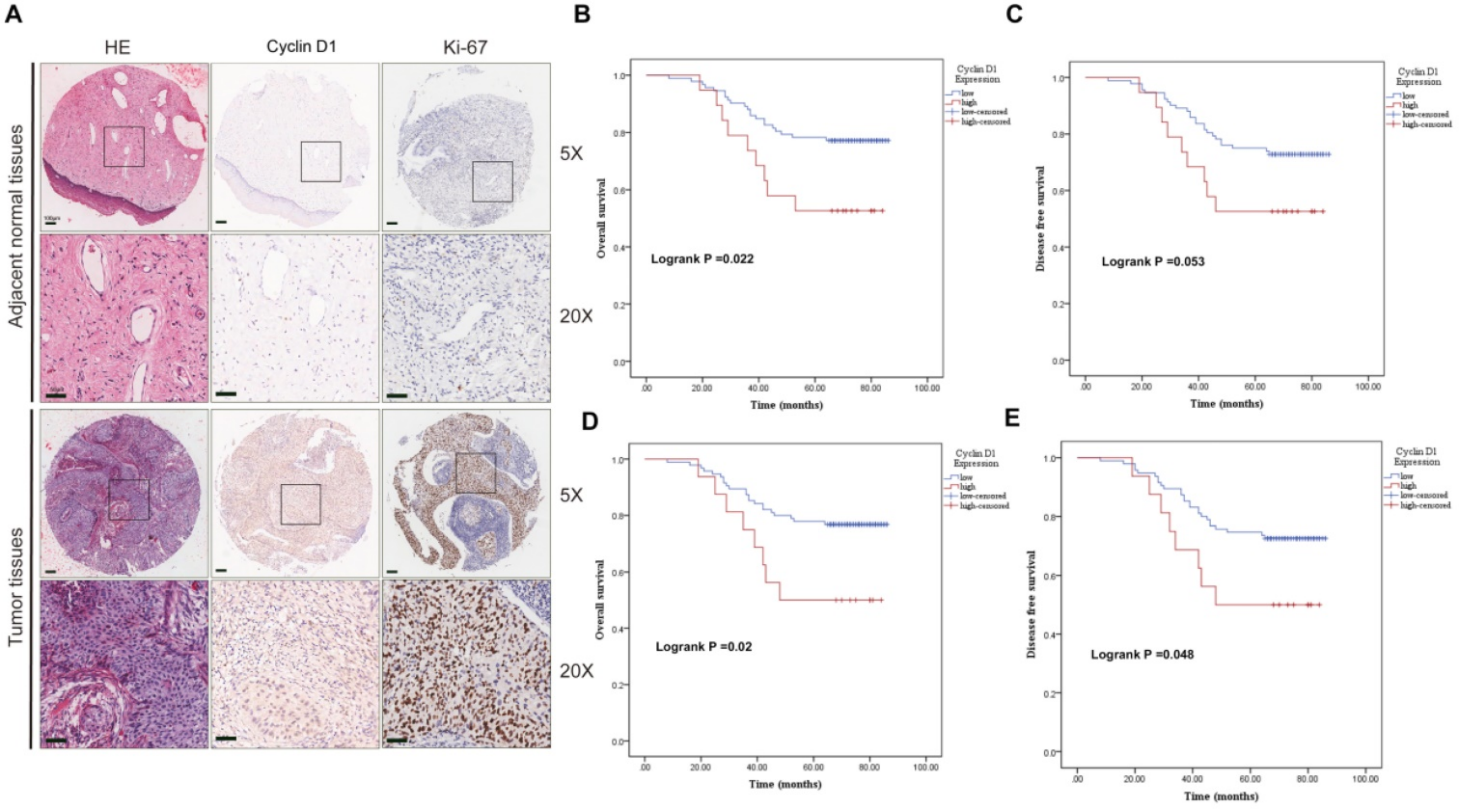
** Upregulated cyclin D1 expression is associated with poor prognosis in cervical cancer TMA cohort. (A)** Representative cyclin D1 and Ki-67 IHC staining patterns in cervical cancer tissue and adjacent normal tissues. Scale bar, 100 µm and 50 µm. **(B and C)** The probability of OS and RFS were analyzed by Kaplan-Meier analysis, according to cyclin D1 staining scores in cytoplasm of the TMA cohort. **(D and E)** The probability of OS and RFS were analyzed by Kaplan-Meier analysis, according to cyclin D1 staining scores in the nucleus of the TMA cohort.

We first evaluated the clinic correlation of cyclin D1 through the cervical cancer TMA cohort. To study the transcription of cyclin D1, we analyzed the expression of cyclin D1 in the cytoplasm and nucleus separately. IHC staining showed cyclin D1 and Ki-67 protein expression in the cervical cancer tissues compared with that in normal tissues (Fig. 1A). Statistical analysis showed that there was no significant difference of cyclin D1 expression between cancer and adjacent tissues (Table 1A cytoplasm, p=0.051. Table 1B nucleus, p=0.125). We found that cervical cancer patients with high cyclin D1 expression had significant lower probabilities of OS (Fig. 1B, p=0.022) and tend to lower DFS (Fig. 1C, p=0.053) in comparison with patients with low cyclin D1 expression in the cytoplasm. Moreover, Kaplan-Meier analysis revealed shorter OS (Fig. 1D, p=0.02) and DFS times (Fig. 1E, p=0.048) in patients with high cyclin D1 expression in the nucleus.

We also analyzed the correlation between cyclin D1 expression and clinicopathologic characteristics. It was showed that the cyclin D1 expression in the cytoplasm was related to age (p=0.014) and grade (p=0.002) (Table 2A), the cyclin D1 expression in the nucleus was also related to age (p=0.04) and grade (p=0.049) (Table 2B). Next, we performed univariate and multivariate analysis of the factors correlated with overall survival of uterine cervix cancer patients with Cox's regression model in the tissue microarray cohort. Age (p=0.013 HR=2.388), FIGO stage (p<0.001, HR=16.975) and high cyclin D1 expression in the cytoplasm (p=0.005, HR=3.494) were significantly associated with poor survival (Table 3A). On the other hand, age (p=0.013 HR=4.599) and FIGO stage (p<0.001, HR=12.332) were also significantly associated with poor survival in the nucleus group (Table 3B). The results suggest that cyclin D1 in the cytoplasm is an independent prognostic factor and that high cyclin D1 expression is associated with poorer survival in cervical cancer.

### Cyclin D1 is down-regulated or up-regulated in C33A model cells

**Figure 2:**
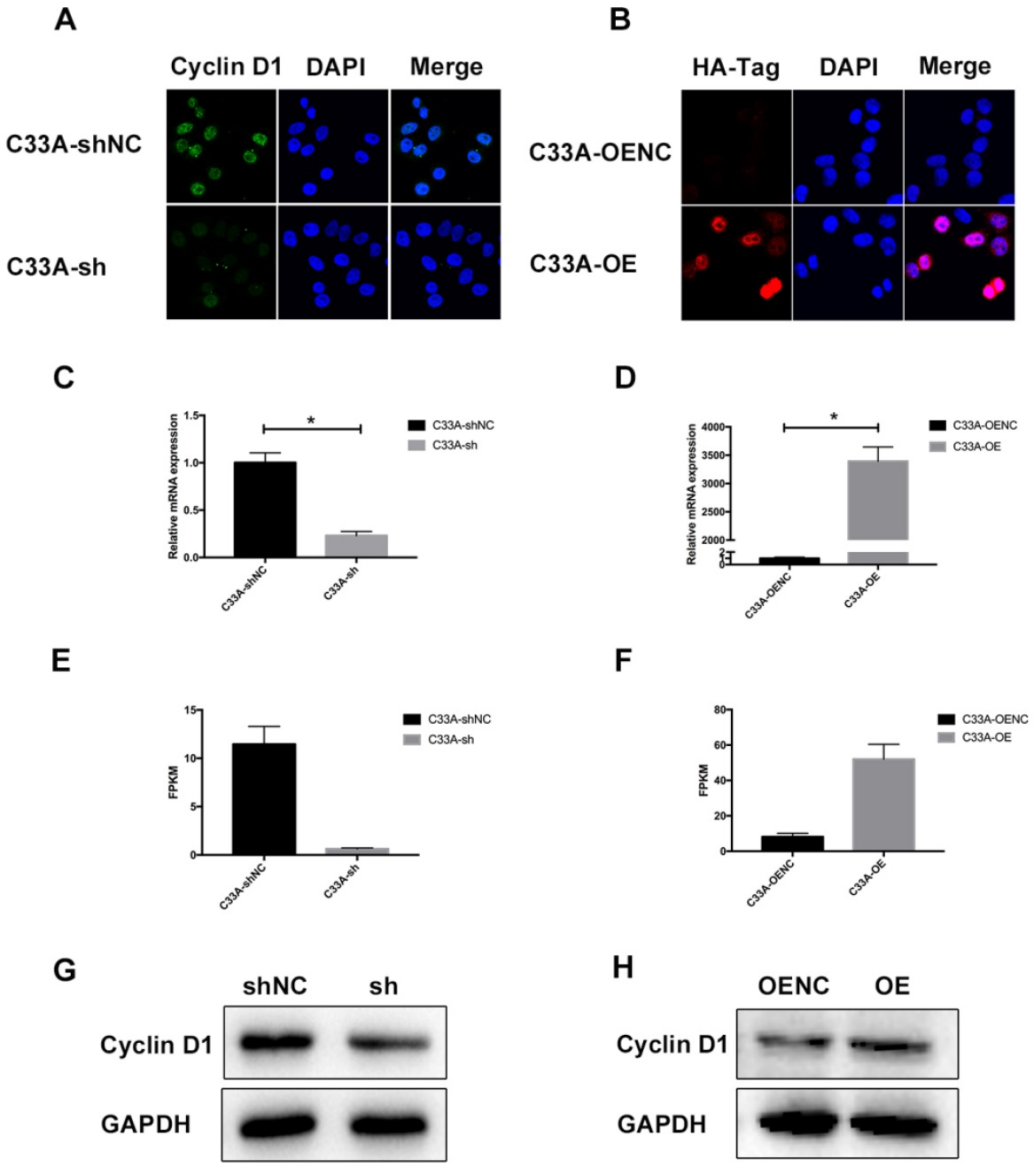
** Cyclin D1 is down-regulated or up-regulated in C33A model cells. (A and B)** Immunofluorescence assay was performed in order to assess the subcellular localization of cyclin D1 in these cells where the green staining represents the cyclin D1 protein and the blue staining represents the nucleolus, and the red staining represents the HA-Tag and the blue staining represents the nucleolus (the magnification is 200X). **(C and D)** The relative mRNA expression of cyclin D1 was decreased in the C33A-sh cells compared to the C33A-shNC cells (P < 0.05), also increased in the C33A-OE cells compared to the C33A-OENC cells (P < 0.05). **(E and F)** The FPKM (Fragments Per Kilobase of transcript per Million fragments mapped) were measured by RNA-seq in C33A model cells, which were Statistically different (P < 0.05). **(G and H)** The results of western blotting demonstrated that we have successfully established the cyclin D1 knock-in C33A and cyclin D1 knock-down C33A.

It was demonstrated that we have successfully established the cyclin D1 knock-in C33A and cyclin D1 knock-down C33A by western blotting (Fig. 2G-H). Immunofluorescence assay was also performed so that we could assess the subcellular localization of cyclin D1 in these cells where the green staining represents the cyclin D1 protein and the blue staining represents the nucleolus, and the red staining represents the HA-Tag and the blue staining represents the nucleolus (Fig. 2A-B). As shown in the Figure, most cyclin D1 protein are localized in the nucleus of cervical cancer cells. Furthermore, we also detected the relative mRNA expression of cyclin D1 by real-time PCR (Fig. 2C-D), and the FPKM (Fragments Per Kilobase of transcript per Million fragments mapped) by RNA-seq (Fig. 2E-F). These results indicated that we successfully established stable transfected cell lines of C33A.

### Analyses of cyclin D1 interaction with the C33A genome

**Figure 3:**
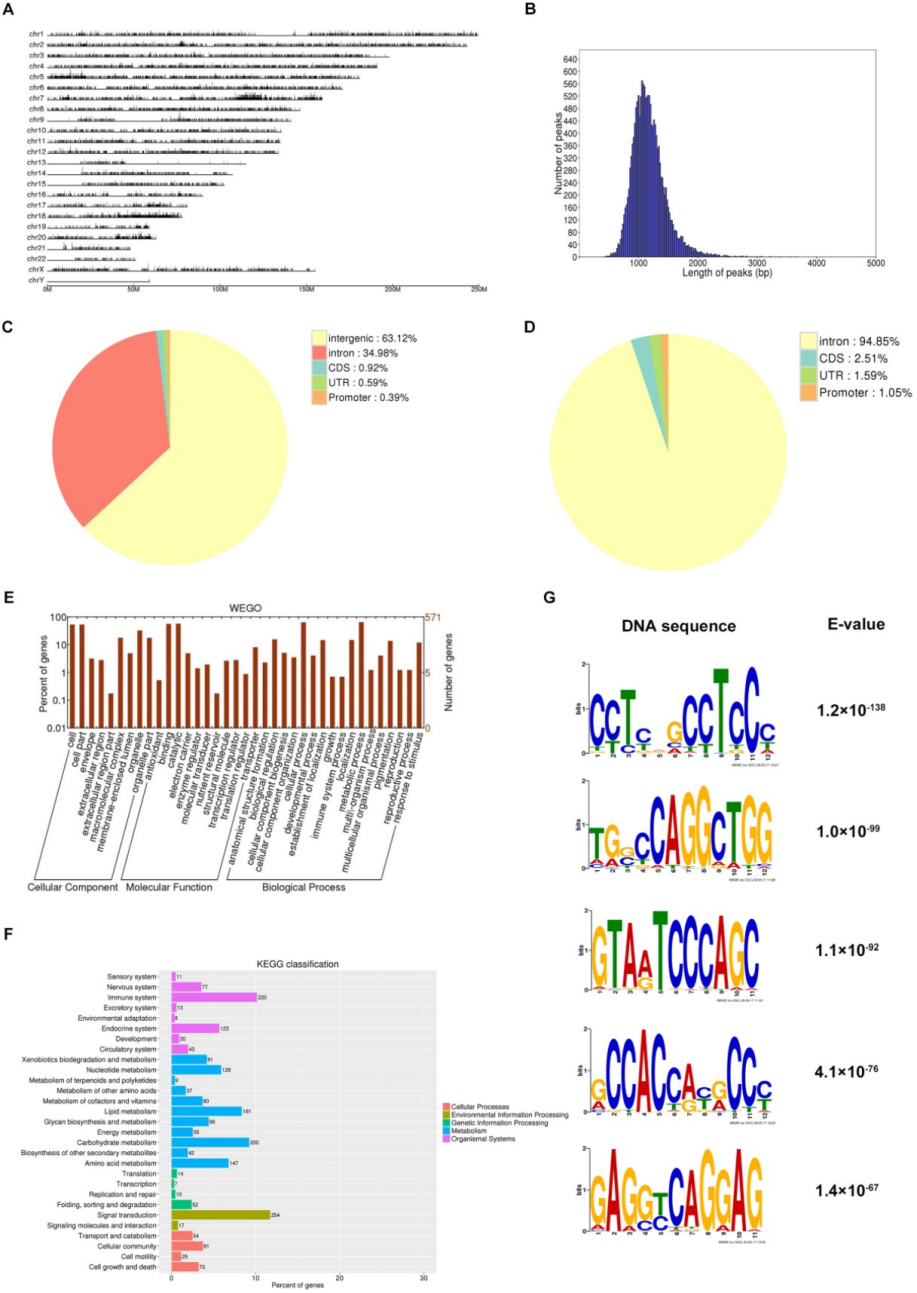
** Analyses of cyclin D1 interaction with the C33A genome. (A)** Cyclin D1 occupies region of abundantly genes from the distribution of potential peak in genome. **(B)** The length of peaks mostly are between 500 and 2000, while focused on nearly 1000 bp. **(C and D)** Most of the binding sites were located in intergenic or intron. **(E and F)** The function of the downstream genes, for example, cellular component, molecular function, biological process; based on the functional annotation analysis, there were a large number of gene sets associated with signal transduction. **(G)** The TF motifs enriched within the interval regions associated with cyclin D1, which suggested that cyclin D1 bound promoter regions with a high content of CpG dinucleotide.

Cyclin D1 occupies region of abundantly genes from the distribution of potential peak in genome (Fig. 3A), and the length of peaks focused on nearly 1000 bp (Fig. 3B). Most of the binding sites were located in intergenic or intron (Fig. 3C-D). Totally we detected 5590 genes involved in binding with cyclin D1 (Supplement 1), and we could find the function of these genes, for example, cellular component, molecular function, biological process; based on the functional annotation analysis, there were a large number of gene sets associated with signal transduction (Fig. 3E-F). We selected five examples of conserved TF motifs enriched within the interval regions associated with cyclin D1, which suggested that cyclin D1 bound promoter regions with a high content of CpG dinucleotide (Fig. 3G).

### Analyses of differential genes in cyclin D1 knock-down C33A cells

**Figure 4:**
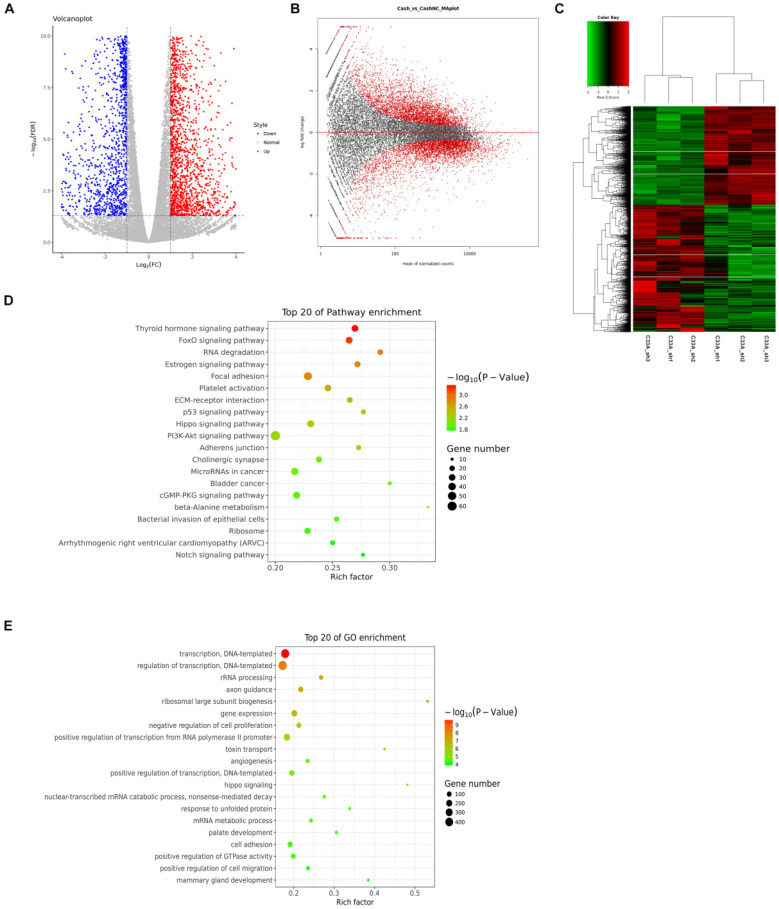
** Analyses of differential genes in cyclin D1 knock-down C33A cells. (A and B)** Each dot represents a gene, the red dots represent up-regulated differentially expressed genes, the blue dots in the figure represent down-regulated differentially expressed genes, and the gray dots represent non-differentiated genes. While the red dots represent differentially expressed genes, and the black dots represent non-differentiated genes. **(C)** The heat maps displayed genes differentially regulated by cyclin D1, the three biological replicates also had good consistency from the hierarchical clustering analyses. **(D)** The differentially expressed genes with KEGG (Kyoto Encyclopedia of Genes and Genomes: systematic analysis of gene function or genomic information database). We selected the top 20 of pathway enrichment which were most significant based on the Rich factor and p value, it was found that these genes involved several important pathways such as PI3K-Akt signaling pathway. **(E)** The top 20 of pathway enrichment by GO-analysis (Gene Ontology), it was found that these genes involved several important pathways such as transcription.

We set fold change ≥2 or fold change ≤1/2 and FDR< 0.05 as a standard for screening differential genes. We detected 2244 genes up-regulation, 1738 genes down-regulation in cyclin D1 knock-down cells by RNA-seq (Supplement 2-3). In Figure 4A, each dot represents a gene, the red dots represent up-regulated differentially expressed genes, the blue dots in the figure represent down-regulated differentially expressed genes, and the gray dots represent non-differentiated genes. In Figure 4B, the red dots represent differentially expressed genes, and the black dots represent non-differentiated genes. The heat maps displayed genes differentially regulated by cyclin D1, the three biological replicates also had good consistency from the hierarchical clustering analyses (Fig. 4C). We analyzed the differentially expressed genes with KEGG (Kyoto Encyclopedia of Genes and Genomes: systematic analysis of gene function or genomic information database). We selected the top 20 of pathway enrichment which were most significant based on the Rich factor and p value, it was found that these genes involved several important pathways such as PI3K-Akt signaling pathway (Fig. 4D). Furthermore, we also selected the top 20 of pathway enrichment by GO-analysis (Gene Ontology), it was found that these genes involved several important pathways such as transcription (Fig. 4E).

### Analyses of differential genes in cyclin D1 knock-in C33A cells

**Figure 5:**
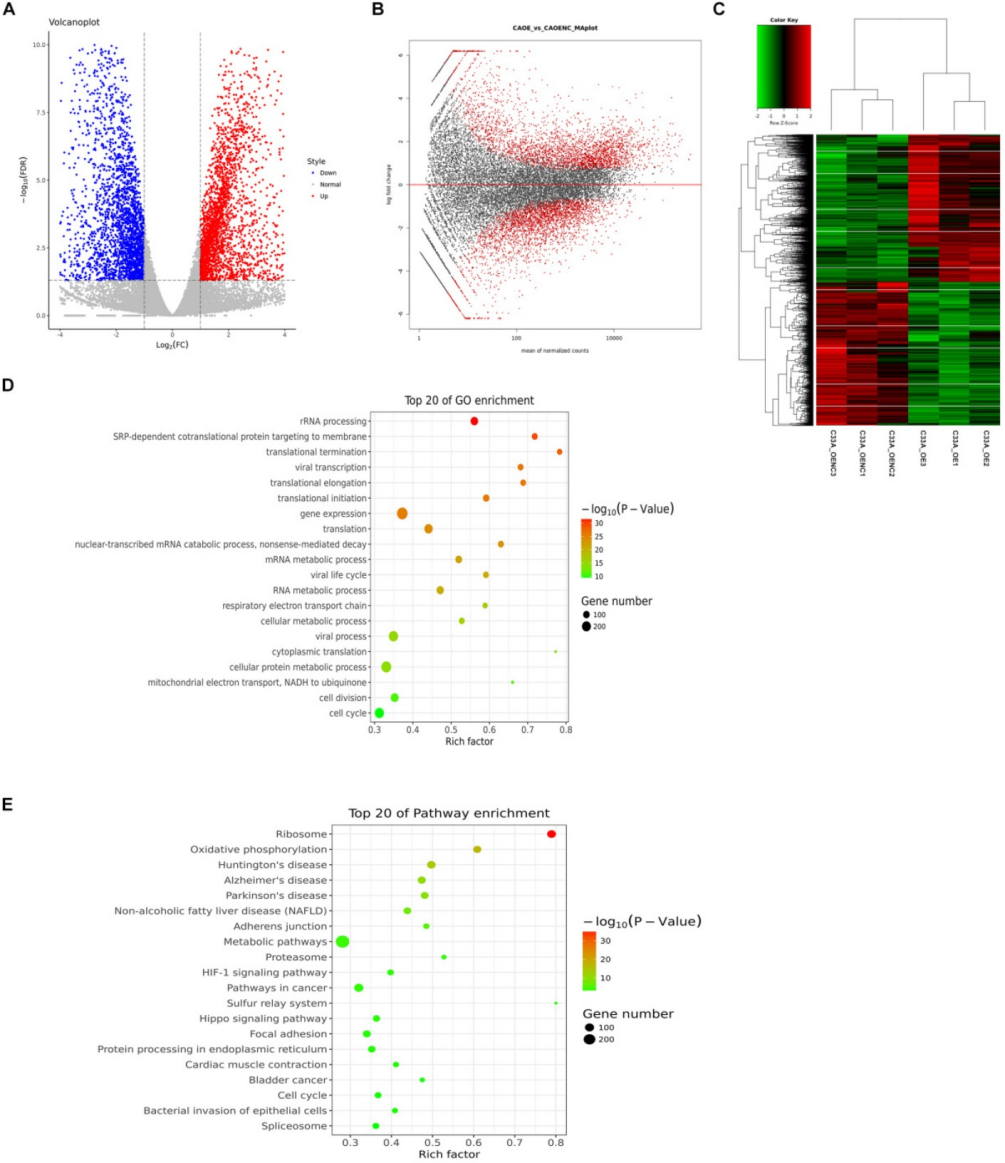
** Analyses of differential genes in cyclin D1 knock-in C33A cells. (A and B)** The down-regulated differentially expressed genes and the up-regulated differentially expressed genes were picked out. **(C)** The heat maps displayed genes differentially regulated by cyclin D1 from the hierarchical clustering analyses. **(D)** The top 20 of pathway enrichment by GO-analysis (Gene Ontology), it was found that these genes involved several important pathways such as gene expression and transcription. **(E)** The differentially expressed genes with KEGG and selected the top 20 of pathway enrichment which were most significant, it was found that these genes involved several important pathways such as metabolic pathways and pathways in cancer.

In the same way, we screened 2811 genes up-regulation, 2727 genes down-regulation in cyclin D1 knock-in cells by RNA-seq (Supplement 4-5). We could find that the down-regulated differentially expressed genes and the up-regulated differentially expressed genes from Figure 5A and 5B. The heat maps displayed genes differentially regulated by cyclin D1, the three biological replicates also had good consistency from the hierarchical clustering analyses (Fig. 5C). What's more, we analyzed the differentially expressed genes with KEGG and selected the top 20 of pathway enrichment which were most significant, it was found that these genes involved several important pathways such as metabolic pathways and pathways in cancer (Fig. 5E). We also selected the top 20 of pathway enrichment by GO-analysis, it was found that these genes involved several important pathways such as gene expression and transcription (Fig. 5D).

### Analyses of combination the ChIP-seq with RNA-seq

**Figure 6:**
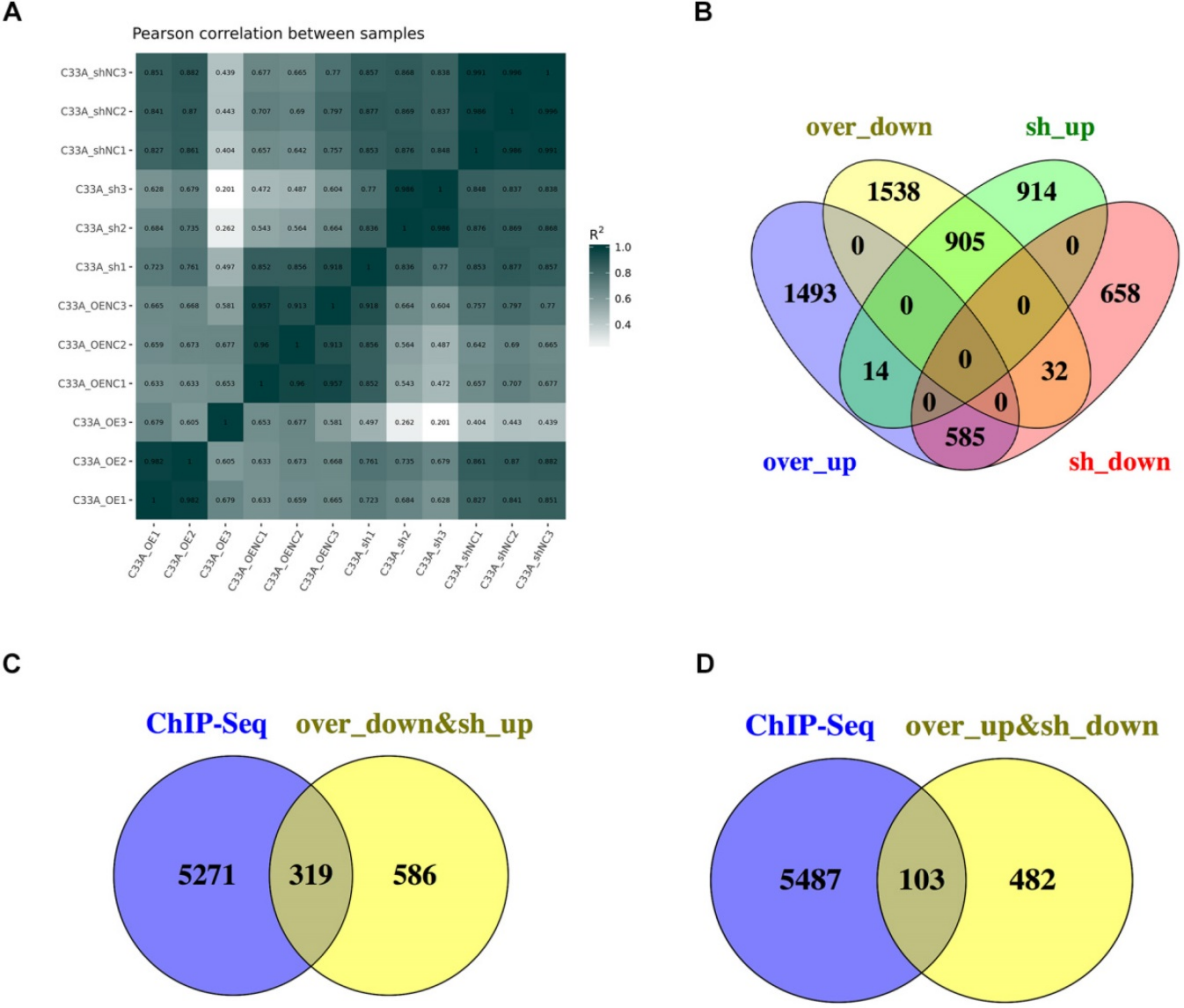
** Analyses of combination the ChIP-seq with RNA-seq. (A)** The Pearson's correlation coefficient, R as an indicator of biological repeat relevance, whose square is closer to 1 represents the correlation is stronger. It was showed that the most biological replicates had strong correlation. **(B)** There were 905 genes in the intersection contained the 2244 genes which is up-regulation in cyclin D1 knock-down cells and 2727 genes down-regulation in cyclin D1 knock-in cells; 585 genes in the intersection contained the 1738 genes which is down-regulation in cyclin D1 knock-down cells and 2811 genes up-regulation in cyclin D1 knock-in cells. **(C and D)** Combination with the ChIP-seq, it suggested that cyclin D1 could activate 103 genes expression and downregulate 319 genes expression by binding with DNA regions.

The Pearson's correlation coefficient, R as an indicator of biological repeat relevance, whose square is closer to 1 represents the correlation is stronger. The most biological replicates had strong correlation from the Figure 6A. To find the downstream genes of cyclin D1, there were 905 genes in the intersection contained the 2244 genes which is up-regulation in cyclin D1 knock-down cells and 2727 genes down-regulation in cyclin D1 knock-in cells; 585 genes in the intersection contained the 1738 genes which is down-regulation in cyclin D1 knock-down cells and 2811 genes up-regulation in cyclin D1 knock-in cells (Fig. 6B). Combination with the ChIP-seq, it suggested that cyclin D1 could activate 103 genes expression and downregulate 319 genes expression by binding with DNA regions (Fig. 6C-D) (Supplement 6).

## Discussion

As we know, nuclear cyclin D1 accumulation results into uncontrolled cell cycle progression and oncogenesis 27. Most researches about cyclin D1 focus on the cell cycle progression, to form a cyclin D-CDK4/CDK6 complex and phosphorylate the Rb protein. Hyperphosphorylation of Rb leads to the release of the E2F family transcription factors and the activation of the transcriptional genes that control cell cycle progression, development, and metabolism 28-30. Some studies reported that cyclin D1 governs DNA damage repair through recruiting DNA repair complexes 21, 31-33.

It remains unclear how the molecular patterns described here are established and maintained, but with the advancement of technology in recent years, it is convenient for us to investigate the genome size, transcription factor binding sites, transcript discovery, and the expression quantification by deep DNA sequencing methods (ChIP-seq and RNA-seq) 34. But we have not found the researches concerning the new function of cyclin D1 on human cancer cells. In order to find the clinic correlation of cyclin D1, we analyzed the correlation of the cyclin D1 expression levels and prognosis of cervical cancer patients in tissue microarray (TMA) cohort. We found that there was no significant difference of cyclin D1 expression between cancer and adjacent tissues, which may due to lack of enough samples. The expression of cyclin D1 in the cytoplasm or nucleus was connected with age and grade (p<0.05). High expression of cyclin D1 in the cytoplasm or nucleus was significantly associated with poor survival. What's more, high expression of cyclin D1 in the nucleus was significantly associated with shorter OS and DFS. Then, in order to find and confirm that the transcriptional role of cyclin D1 in cervical cancer, we performed the ChIP-seq and RNA -seq on the C33A cells. We found that cyclin D1 was localized in the nucleus by immunofluorescence staining, which occupied abundantly DNA sequences. Our finding confirmed the finding of Bienvenu, F et al. mentioned that during mouse development cyclin D1 occupies promoters of abundantly expressed genes as a transcriptional role, in this research they found the cyclin D1-bound promoters were highly enriched for CpG dinucleotide (P<1X10^-15^) 20, because it has been proved that genes that are highly expressed in many tissues were shown to have a high content of CpG dinucleotides in their promoter regions 35. Then, we found the expression of abundantly genes changed as the expression of cyclin D1 changed, which suggested that cyclin D1 could serve both to activate and downregulate gene expression. To find out the genes whose expression changed as a result of cyclin D1-bound promoters, we analyzed the data of ChIP-seq and RNA seq. 422 genes were distinguished, of which 103 genes were positive correlation with the expression of cyclin D1 and 319 genes were negative correlation with the expression of cyclin D1.

Here, we found the clinic correlation of cyclin D1 which related to prognosis, and then conducted a genome-wide analysis of cyclin D1 binding in the context of local chromatin by using ChIP-seq analysis. The intrinsic DNA sequence-specific binding of cyclin D1 can be conjectured by the motifs. But it is still not very clear how the cyclin D1 protein is recruited to DNA, a study proposed it could be recruited to DNA through sequence-specific binding proteins 22, 33. The main discovery of this work is the demonstration that cyclin D1 plays a transcriptional function in cervical cancer cell line C33A by acting at gene promoters. Although our mechanistic analyses focused on the downstream genes, it remains to be seen whether this transcriptional function contributes to cellular component, molecular function and biological process, such as PI3K signaling pathway. It will be also of interest to determine whether this function of cyclin D1 contributes to oncogenesis and tumor development.

## Conclusions

In conclusion, our investigation improves the study about the clinic correlation and function of cyclin D1, because it not only has the function of regulating the cell cycle and it is not necessary for pRB-negative cancer cells to proliferation such as HeLa 36, 37. We find the downstream genes are highly enriched in Gene Ontology categories and involve in diverse cellular functions via KEGG classification, including translation, replication and repair, signal transduction, cell growth and death, which means that cyclin D1 may be a key protein during oncogenesis and tumor development. It shows that cyclin D1 may be a potential therapeutic target in cancer therapy. However, the mechanisms of how these genes specifically affect the biological behavior of tumors and their development still require further research.

## Supplementary Material

Supplementary data.

## Figures and Tables

**Table 1A:** Differential expression of Cyclin D1 in cancer and adjacent tissues

	n	Cyclin D1 expression		Chi-square value	*p* value
		High	Low		
Cancer	111	18	93	0.355	0.551
Adjacent tissues	26	3	23		

**Table 1B:** Differential expression of Cyclin D1 in cancer and adjacent tissues

	n	Cyclin D1 expression		Chi-square value	*p* value
		High	Low		
Cancer	111	16	95	2.359	0.125
Adjacent tissues	26	7	19		

**Table 2A:** Correlation between Cyclin D1 expression and clinicopathologic characteristics

Variables	Cyclin D1 expression		Total	χ^2^	*p* value
	low	high			
**Age (year)**				6.091	0.014
≤42	37	2	39		
>42	55	17	72		
**Grade**				9.367	0.002
I/II	9	7	16		
III	67	9	76		
**T stage**				0.496	0.481
T1	50	12	62		
T2/T3/T4	42	7	49		
**N stage**				0.068	0.794
N0	75	15	90		
N1	17	4	21		
**FIGO stage**				0.496	0.481
I	50	12	62		
II/III/IV	42	7	49		

**Table 2B:** Correlation between Cyclin D1 expression and clinicopathologic characteristics

Variables	Cyclin D1 expression		Total	χ^2^	*p* value
	low	high			
**Age (year)**				4.203	0.04
≤42	37	2	39		
>42	58	14	72		
**Grade**				3.859	0.049
I/II	11	5	16		
III	67	9	76		
**T stage**				0.260	0.61
T1	54	8	62		
T2/T3/T4	41	8	49		
**N stage**				1.853	0.173
N0	79	11	90		
N1	16	5	21		
**FIGO stage**				0.260	0.61
I	54	8	62		
II/III/IV	41	8	49		

**Table 3A:** Univariate and multivariate analyses of the factors correlated with overall survival of uterine cervix cancer patients

Variables	Univariate analysis			Multivariate analysis		
	*p* value	HR	95%CI	*p* value	HR	95%CI
Expression	0.027	2.415	1.105-5.279	0.005	3.494	1.453-8.4
Age	0.002	6.453	1.967-21.164	0.013	4.577	1.374-15.25
Grade stage	0.233	2.068	0.627-6.821			
FIGO stage	<0.001	13.525	4.741-38.583	<0.001	16.975	4.51-63.897
T stage	<0.001	13.525	4.741-38.583	NA	NA	NA
N stage	<0.001	5.363	2.689-10.7	0.361	1.463	0.647-3.306

**Table 3B:** Univariate and multivariate analyses of the factors correlated with Overall survival of uterine cervix cancer patients

Variables	Univariate analysis			Multivariate analysis		
	*p* value	HR	95%CI	*p* value	HR	95%CI
Expression	0.025	2.525	1.123-5.681	0.14	1.966	0.801-4.827
Age	0.002	6.453	1.967-21.164	0.013	4.599	1.374-15.397
Grade stage	0.233	2.068	0.627-6.821			
FIGO stage	<0.001	13.525	4.741-38.583	<0.001	12.332	3.44-44.21
T stage	<0.001	13.525	4.741-38.583	NA	NA	NA
N stage	<0.001	5.363	2.689-10.7	0.258	1.609	0.705-3.671

## Abbreviations

CDK: cyclin-dependent kinase
pRb: retinoblastoma gene product
ChIP-seq: chromatin immunoprecipitation followed by high-throughput sequencing
RNA-seq: RNA-sequencing
HA-Tag: human influenza hemagglutinin tag
PCR: polymerase chain reaction
FPKM: fragmentsper kilobase of transcript per million fragments mapped
TF motifs: transcription factor motifs
KEGG: Kyoto Encyclopedia of Genes and Genomes
